# Alumina Matrix Composites with Non-Oxide Nanoparticle Addition and Enhanced Functionalities

**DOI:** 10.3390/nano5010115

**Published:** 2015-01-27

**Authors:** Dušan Galusek, Dagmar Galusková

**Affiliations:** 1 Joint Glass Centre of the Institute of Inorganic Chemistry, Slovak Academy of Sciences, Alexander Dubček University of Trenčín, Študentská 2, 91150 Trenčín, Slovak Republic; E-Mail: dagmar.galuskova@tnuni.sk; 2 Faculty of Chemical and Food Technology, Slovak University of Technology in Bratislava, Vazovova 5, 81243 Bratislava, Slovak Republic

**Keywords:** alumina-based nanocomposites, SiC, CNT, CNF, mechanical properties, functional properties

## Abstract

The addition of SiC or TiC nanoparticles to polycrystalline alumina matrix has long been known as an efficient way of improving the mechanical properties of alumina-based ceramics, especially strength, creep, and wear resistance. Recently, new types of nano-additives, such as carbon nanotubes (CNT), carbon nanofibers (CNF), and graphene sheets have been studied in order not only to improve the mechanical properties, but also to prepare materials with added functionalities, such as thermal and electrical conductivity. This paper provides a concise review of several types of alumina-based nanocomposites, evaluating the efficiency of various preparation methods and additives in terms of their influence on the properties of composites.

## 1. Introduction

Many ceramic materials used in engineering suffer from inherent brittleness and generally inferior mechanical properties compared to metals. Concentrated efforts in the last couple of decades have therefore been aimed at identification of methods that would result in better ceramics, ceramic-matrix composites (CMCs) chief among them. Since the pioneering work of Niihara in the 1990s [1], the addition of nano-particles or whiskers of a second phase was considered to be one of the most promising ways of improving the mechanical properties of polycrystalline alumina-based ceramics. Extensive literature published on the topic indicates that the addition of silicon carbide particles (SiC_p_) or whiskers (SiC_w_) to polycrystalline alumina improves strength [1,2,3,4,5,6], fracture toughness [6,7,8], wear resistance [9,10,11], and creep resistance [12,13,14], compared to monolithic polycrystalline alumina. However, despite the tremendous efforts documented by thousands of research papers published on the topic, the so-called “nanocomposites” have generally remained a topic of academic research, failing to make their breakthrough to large-scale production. The problems with homogeneous distribution of nanoparticles, and related problems with reproducible preparation of materials with improved mechanical properties, can be considered as a chief obstacle. Renewed interest in CMCs with alumina matrix was observed with the invention, and commercial availability, of carbon nanotubes (CNT) and nanofibers (CNF). The intrinsic properties of the CNTs are impressive. The theoretical mechanical strength of 30 GPa calculated for both the single-wall (SWCNT) and multi-wall carbon nanotubes (MWCNT), together with an extremely high Young’s modulus (1 and 1–1.8 TPa, respectively) [15,16,17,18], immediately made them first-class candidates for preparation of CMCs with significantly improved mechanical properties. Other properties of the CNTs are even more impressive: the “metallic” character of SWCNT with armchair structure results in high electrical conductivity, which combines with extremely high thermal conductivity. The theoretically calculated values of the latter range between 2800 and 6000 W·m^−1^·K^−1^, making them the best heat conductors known [19]. Attempts to use the CNTs for preparation of CMCs with added functionalities, such as high thermal and electrical conductivity, then came as no surprise, providing added value to expected improvement of mechanical properties. This paper is an attempt to summarize the preparation of CMCs with polycrystalline alumina matrix, with improved mechanical properties and added functionalities, supplemented by critical evaluation of reported achievements.

## 2. Preparation of Nanocomposites

### 2.1. Homogeneous Distribution of Nanoparticles

Homogeneous mixing of both the matrix and reinforcing phases and even distribution of the reinforcing phase in ceramic matrix are prerequisites for achievement of the desired properties in CMCs. This is of particular importance if nanoparticles are used as the reinforcing phase, due to their extremely high specific surface and, hence, intrinsically high tendency to agglomeration. With the use of nanotubes or nanofibers, the problem is even more aggravated by their shape, leading to formation of severely entangled bundles, which are extremely difficult to de-agglomerate. The following section therefore deals with the ways devised to ensure de-agglomeration and/or homogeneous distribution of nanoparticles in a ceramic matrix.

#### 2.1.1. Al_2_O_3_-SiC

The traditional (so called powder) route consists of mixing the alumina and SiC nano-powders in a suitable aqueous or non-aqueous media, drying, and green body shaping. This method has several serious drawbacks. It is extremely difficult to prevent agglomeration of the SiC nanoparticles and to ensure homogeneous mixing of SiC with Al_2_O_3_. Drying of suspensions is another source of agglomeration, which results in uneven sintering and void and crack formation in the course of densification. Walker *et al.* [20] investigated different drying methods of composite suspensions, and concluded that a freeze-drying technique can be successfully applied in order to avoid the formation of hard agglomerates. A pH adjustment can also be used to induce flocculation of the slurry, preventing segregation and agglomeration of the silicon carbide particles [21].

In order to prevent the problems caused by mixing of SiC and Al_2_O_3_ powders, composite powders can be synthesized by carbothermal reduction of a mixture of silica and alumina or of natural aluminosilicates like kaolinite [22], kyanite [23], and andalusite [24]. Suitable adjustment of reaction conditions (chemical composition of starting mixture or mineral, reaction temperature, source and partial pressure of C, dwell time at reaction temperature, or presence of impurities or catalysts) allows the control of the content and morphology of SiC fraction in the mixed powder. Sol-gel synthesis can also be applied for preparation of alumina-SiC nanopowders: The SiC nanopowder is dispersed in a suitable liquid medium to create a stable suspension and mixed with a liquid alumina precursor, such as Al_2_O_3_ [25] or AlCl_3_ solution [26,27]. After gelation and drying, the xerogel containing SiC nanoparticles is calcined, crushed, sieved, and finally used for preparation of nanocomposites. Other attempts to prepare the nanocomposite powders use more exotic techniques, such as a Teflon-activated self-propagating aluminothermic reaction (Equation (1)) [28]:

4Al + SiO_2_ + 3C→3SiC + 2Al_2_O_3_ (1)

or thermal-gradient chemical vapor infiltration of SiC porous preforms with a gaseous mixture of AlCl_3_, H_2_, and CO_2_, resulting in inhomogeneous distribution of constituent phases [29].

The most promising among the non-traditional routes of preparation of nanocomposite powders is the so-called “hybrid” route, in other words the route utilizing the ceramization of organosilicon polymeric precursors of SiC, typically polycarbosilanes [30,31,32,33,34]_._ This is usually based on coating the alumina particles with a dissolved polymer, followed by drying, cross-linking of the polymer, pyrolysis, and densification. The method allows preparation of alumina-based nanocomposites with ultrafine particles of SiC (~12 nm) evenly distributed in the alumina matrix both at intra- [30,31] and inter-granular [32] positions. The agglomeration in the course of drying is usually avoided by application of advanced drying techniques like freeze-drying and freeze granulation, or by the use of wet shaping techniques like slip casting, tape casting, and pressure filtration. An alternative shaping route adapted from processing of polymers is represented by axial pressing of polymer-coated powders at elevated temperatures of 300–400 °C (called also warm pressing), yielding a dense green body with alumina particles embedded in a matrix of a highly cross-linked preceramic polymer, which is transformed, upon heating in inert atmosphere (Ar), to SiC [34].

The preceramic polymers can be also used for infiltration of pre-sintered porous alumina matrix. For that purpose either liquid polymers are used (e.g., poly-allylcarobosilanes), or solid polymers are dissolved in a suitable solvent (e.g., cyclohexane). After infiltration the solvent is evaporated, and the polymer transformed to SiC *in situ*, entirely avoiding the problems associated with de-agglomeration. The size and distribution of SiC nanoparticles are then readily controlled by the size and distribution of open pores in the alumina matrix, which is adjusted by selection of the alumina powder, the shaping technique used, and the conditions of pre-sintering [35].

#### 2.1.2. Al_2_O_3_-CNT, CNF

In principle, there exist two different attitudes aimed at achievement of even and homogeneous distribution of CNTs in the composites with polycrystalline alumina matrix. The first one relies on the ability to disentangle the nanotube bundles, separate the individual nanotubes by chemical or mechanical means (or their combination), and then mix the nanotubes with alumina powder or suspension. Mechanical de-agglomeration of CNTs requires an energetic method of separation, such as shear mixing, ball milling, ultra-sonication [36], gas purging sonication (combination of sonication with simultaneous purging with nitrogen gas), or others in a suitable liquid medium, ethanol being the most frequently used [36,37]. A suitable dispersant, such as sodium dodecyl sulfate (SDS), is often used to stabilize the suspension [37]. The attitude often requires previous chemical “functionalization” of nanotubes, *i.e.*, their pre-treatment in a mixture of inorganic acids at elevated temperature. The process involves heating nanotubes for several hours in a mixture of nitric and sulfuric acid at temperatures often exceeding 100 °C [38]. This rather harsh treatment has two outcomes: first, the metallic catalyst, as the impurity resulting from synthesis of the nanotubes, is thoroughly removed. Second, polar functional groups (such as C=O, and C–O–H) are formed at the surface of the nanotubes, changing their intrinsically hydrophobic nature to hydrophilic, and creating reaction centers facilitating attachment of dispersant molecules [39]. However, as an undesired byproduct, the treatment often leads to deterioration and defect formation at the surface of nanotubes. A typical procedure then involves dropwise addition of alumina suspension into a stabilized and vigorously stirred suspension of the nanotubes, and its homogenization by ball milling or sonication [36]. The composite suspension is then dried in a way that prevents re-agglomeration of the nanotubes; among the possible ways, freeze granulation has been recently reported as the most successful [40]. The composite powder is then consolidated by a suitable forming method and densified, as will be discussed in more detail in Section 2.2. One of the alternatives is represented by preparation of alumina-coated nanotubes by hydrothermal crystallization, thus achieving better compatibility and stronger bonding of the nanotubes with the alumina matrix [41].

The second approach is based on the ability of nanotubes to grow from a suitable gas atmosphere on a metallic (Ni, Co, or Fe) precursor *in situ*, *i.e.*, directly on alumina powder particles or within a porous alumina matrix. Various solutions of salts of the metal catalysts are used to disperse Fe, Ni, or Co onto ceramic supports [42,43,44]. A typical procedure includes preparation of a solution of suitable nitrates with alumina powder or an alumina precursor, such as Al(NO_3_)_3_·9H_2_O with a mixture of citric acid and urea used as fuel. The mixture is heated until ignition of the fuel: in this way alumina powder homogenously doped with the metallic catalyst is prepared by combustion synthesis [45,46]. After calcination the powder is placed in a reaction furnace or a CCVD chamber and heated up to 1000 °C in a mixed H_2_/CH_4_ atmosphere. Carbon required for the growth of nanotubes originates from catalytic decomposition of methane on transition metal nanoparticles formed by combustion synthesis [47]. In this way the difficulties related to de-agglomeration and homogeneous dispersion of the nanotubes in the alumina matrix are avoided, and composite green bodies can be readily prepared.

### 2.2. Densification

#### 2.2.1. Al_2_O_3_-SiC

**Figure 1:**
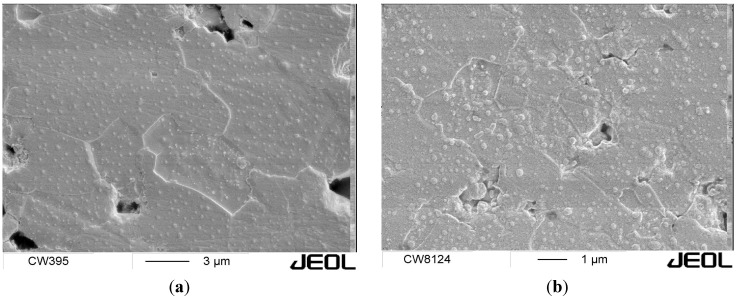
Microstructure refinement observed in Al_2_O_3_-SiC nanocomposites with increasing volume fraction of SiC nanoparticles: (**a**) 3 vol% SiC; (**b**) 8 vol% SiC. The nanocomposites were sintered without pressure at 1750 °C from a green body prepared by warm pressing of poly(allyl)carbosilane-coated alumina powder [34].

All available consolidation techniques yield composite green bodies with relatively high porosity (usually around 50%) and more or less homogeneously distributed reinforcing SiC particles, which ideally contain no defects or agglomerates. However, the nature of the composite powders’ preparation results in a microstructural arrangement where the SiC nanoparticles are located at the interfaces between alumina grains, acting as efficient obstacles to densification, and impeding grain boundary motion through a pinning mechanism. The use of pressure-assisted sintering techniques, such as hot pressing, spark plasma sintering, or application of high sintering temperatures (typically between 1700 and 1850 °C) in the case of pressureless sintering, are usually required for preparation of fully dense Al_2_O_3_-SiC nanocomposites. However, the high temperatures accelerate grain boundary motion, resulting in coarse grained microstructure. In addition, the SiC particles at intergranular positions are swallowed by fast moving grain boundaries, creating inclusions inside the alumina matrix grains (Figure 1). The resulting microstructure is coarse grained, with the size of alumina matrix grains in the range of about 5 μm or more [48]. Problems encountered during the pressureless sintering can be, at least partially, solved by the use of sintering additives. Fully dense alumina–SiC nanocomposites were prepared by free sintering followed by gas pressure sintering of the mixture of submicrometer alumina and SiC powder doped with 0.1 wt% MgO and/or Y_2_O_3_ [49]. The addition of other liquid-forming additives, such as MnO_2_.SiO_2_ and CaO.ZnO.SiO_2_, allows densification of Al_2_O_3_-SiC green compacts by free sintering at temperatures as low as 1300 °C, but the resulting microstructures are usually coarse-grained. Abnormally large alumina grains are present, and the distribution of SiC particles is uneven [50]. The ability to densify the nanocomposites by pressureless sintering is limited by the content of SiC nanoparticles, which usually does not exceed 10 vol%. Fully dense composites with higher SiC content (>20 vol%) are prepared by combination of pressureless sintering with hot isostatic pressing (sinter-HIP) [51]. In this case a pre-sintered alumina compact is multiple pressure-infiltrated by a polymer SiC precursor. The precursor is then pyrolyzed in Ar, and sintered without pressure in inert gas or vacuum until the porosity closes, *i.e.*, after the relative density of about 92%–95% is achieved. Then the temperature is increased up to 1750 °C, and a high pressure (up to 150 MPa) of inert gas (Ar) is applied to achieve complete densification. Such a consolidation method benefits from the advantages of both pressureless and high-pressure processes and is a suitable alternative for mass production of ceramic nanocomposite components with complex shapes.

#### 2.2.2. Al_2_O_3_-CNT, CNF

If the pinning effect of intergranular SiC particles is a serious obstacle for achieving high relative density in Al_2_O_3_-SiC nano-composites, the same applies, in an even more serious way, to the composites with added carbon nanotubes or carbon nanofibers. Due to their high aspect ratio, high specific surface, and chemical incompatibility with the surrounding alumina matrix, the CNTs act in two different ways: (a) they impair densification, with resulting decrease of sintered densities; and (b) they reduce the size of alumina matrix grains by sharply decreasing the grain boundary mobility through highly efficient grain boundary pinning [52,53,54]. Another factor contributing to low sintered densities is the presence of CNT clusters at the grain boundaries, as a result of imperfect de-agglomeration. The agglomerates act both as solid obstacles at grain boundary interfaces, which impair densification and grain boundary mobility even more efficiently than individual nanotubes, but also as solid un-sinterable porous objects, which decrease the sintered density [55,56]. It therefore stands to reason that the densification of carbon nanotubes containing nanocomposites is difficult, with difficulties growing with increasing content of CNT and CNFs. Although numerous attempts have been reported on pressureless sintering of CNT-reinforced alumina composites, they were seldom successful. The sintering temperatures usually range between 1200 and 1800 °C in air [36] or, more typically, in inert atmosphere (usually Ar), which is used to avoid oxidation damage and burning out of the CNTs from ceramic matrix. The difficulties related to densification of CNT-containing composites are demonstrated for example by the works of Zhang *et al.* [55] and Rice *et al.* [56], who found a sharp drop of relative density from 98.5% in the composite containing 1 wt% of CNTs to less than 95% when the CNT content increased above 3 vol%. However, in order to avoid the reactions between the alumina matrix and the CNTs, which could lead to CNT loss, the maximum temperature of sintering has to be kept below 1550 °C [56]. Similar results were obtained by Yamamoto *et al.* [57], who sintered Al_2_O_3_-MWCNT composites with up to 3 vol% of MWCNTs with various mechanical properties at 1400 °C in a flowing 95% Ar/5% H_2_ atmosphere. The authors observed a monotonous decrease of relative density with increasing content of the MWCNT in all studied composites, from 99% at 0.5 vol% of the MWCNT to approximately 94% when the MWCNT contents approached 3 vol%.

More attention has therefore been paid to pressure assisted techniques, including hot pressing (HP), hot isostatic pressing (HIP), and spark plasma sintering (SPS). Although the pressure-assisted techniques can inflict some mechanical damage on CNTs, they are usually considered more suitable due to the following reasons: (1) they allow the use of lower temperatures, and shorter times of densification, thus reducing the thermal and oxidation damage of the nanotubes; and (2) they facilitate the achievement of higher relative densities, and through reducing the time and temperature of sintering also produce a finer grained final microstructure in the composite. Several authors successfully densified the Al_2_O_3_-CNT nanocomposites by hot pressing in a graphite die at an applied mechanical pressure up to 40 MPa, and temperatures ranging from 1600 to 1800 °C [39,52,58,59]. The method, if properly conducted, facilitates the preparation of the Al_2_O_3_-MWCNT nanocomposites containing up to 3 vol% of the nanotubes, and with the relative density at the level, or exceeding 99%. Spark plasma sintering has been favored in the last few years due to its ability to apply very high heating rates and achieve complete densification in minutes, rather than hours as in HP [60,61]. However, for this technique high residual porosity is also typical in the composites with high CNT contents. Ahmad *et al.* [62], spark plasma sintered CNT–alumina composites with a CNT content ranging between 1.1 and 10.4 vol% for 3 min at 1400 °C and an axial pressure of 50 MPa, and observed a marked drop of relative density from 98.5% (1.1% MWCNT) to 92% (10.4% MWCNT). Inability to achieve complete densification, together with imperfect de-agglomeration and poor control over the interfaces between CNTs and Al_2_O_3_, are thus major obstacles to unambiguous evaluation of the influence of CNT addition on functional and mechanical properties of CNT-containing CMCs.

## 3. Room and High-Temperature Mechanical Properties

### 3.1. Al_2_O_3_-SiC

Significant attention has been attracted to Al_2_O_3_-SiC composites by a pioneering work of Niihara, whose concept of nanocomposites (addition of nano-sized particles of SiC to microcrystalline alumina matrix) allowed preparation of the Al_2_O_3_-SiC_p_ materials with the flexural strength exceeding 1 GPa and increased fracture toughness [1]. Despite tremendous effort, the reason for such an improvement remains unclear. Niihara himself suggests that the strengthening arises due to the refinement of the microstructural scale from the order of the alumina matrix grain size to the order of the SiC interparticle spacing, thus reducing the critical flaw size. Strengthening can also be explained by the toughening effect caused by crack deflection due to the tensile stresses developed in alumina grains around the SiC particles as a result of thermo-elastic mismatch [1]. However, the observed toughness increase is not sufficient to account for observed strengthening. Many authors also failed to reproduce the results reported by Niihara. As a result, there exists no general agreement on the existence of the so-called “nanocomposite” effect, and alternative explanations of the observed strengthening, often related to processing or machining effects, are provided. These include: (1) elimination of processing flaws and suppression of grain growth in nanocomposites [63,64]; (2) elimination of grain pull-out during surface machining with resulting enhanced resistance to surface defect nucleation [10]; (3) generation of high level surface compressive residual stresses during machining [4,5,65]; or (4) increased tendency of nanocomposites to crack during annealing [4,66].

Even though the increase of fracture toughness in nanocomposites was never reported to be high, the results achieved by various authors are still more controversial. While several papers report modest increase of toughness of nanocomposites over that of unreinforced ceramics [67], others do not find any appreciable change [5,63,68], or even report a reduction in the fracture toughness depending on the measurement technique. One of the reasons may be that the mechanical properties of nanocomposites are strongly influenced by slight changes of the processing route [3]. Another factor, whose role is not clear, is the role of intra- and intergranular SiC particles in defining the mechanical properties, especially fracture toughness. Unlike monolithic aluminas, which usually fail by grain boundary fracture, cracks in Al_2_O_3_-SiC nanocomposites follow an almost entirely transgranular path. Some authors suggest that the change of the fracture mode is caused by tensile tangential stresses in alumina matrix grains around intragranular SiC inclusions. Combined with radial, grain boundary strengthening compressive stresses, the cracks are turned into alumina matrix grains so that they follow a transgranular path, being attracted by intragranular SiC inclusions. The increase of fracture energy is not observed, as the increase of toughness resulting from the change of fracture mode from intergranular to transgranular is compensated by the crack passing through tensile stress fields between second phase and matrix particles [5]. However, Jiao and Jenkins, who performed a detailed analysis of crack propagation in nanocomposites, observed no such attraction, not even in a crack moving very close to an intragranular SiC particle [69]. Other authors consider the ratio of volume fractions of intra- and intergranular SiC as an important parameter, which influences the fracture toughness of nanocomposites. The cracks are attracted to intergranular particles due to the formation of tensile residual stress fields around particles, and perpendicular to adjacent boundaries. This mechanism is expected to increase crack deflection length, at least to a certain extent, and thus to contribute to toughening of the nanocomposite [67]. Significant toughening with a steep R-curve is achieved only by the addition of SiC whiskers [6,8]. High fracture toughness also results in markedly improved thermal shock resistance of whisker-reinforced alumina composites [7,70].

Wear resistance is probably the only room temperature mechanical property where unambiguous improvement in comparison to monolithic alumina is observed [71]. The published data on wear of Al_2_O_3_-SiC nanocomposites and the monolithic alumina report that erosion resistance is more than three times higher [9,72,73], and note a reduction of the dry sliding wear rate [74] of composites with respect to the monolithic alumina with comparable grain size. Addition of SiC nanoparticles into polycrystalline alumina also produces a noticeable improvement in surface quality during lapping and polishing [10,75]. This is considered to be the result of a reduction of grain pullout during grinding and polishing, which, in turn, is believed to be the consequence of an altered method of fracture—from intergranular in monolithic alumina to transgranular in nanocomposites [1]. There exist various explanations for the observed change of the fracture mode, ranging from the strengthening of grain boundaries [76,77] and crack deflection from grain boundaries into the interior of alumina grains by thermal residual stresses around intragranular SiC particles [67], through changes in surface flaw population, to the presence of surface residual stresses [78]. Todd and Limpichaipanit suggest that the role of SiC in nanocomposites with high SiC volume fractions (10 vol%) is in suppression of brittle fracture of alumina by blocking the formation of long twins and dislocation pileups, which are thought to be responsible for crack initiation by intragranular SiC particles (*i.e.*, a form of slip homogenization). They also suggest that the reason for the observed change of fracture mode from intergranular in monolithic alumina to transgranular in SiC-containing composites (including those with added micrometer-sized SiC particles) can be sought in the change of the system’s chemistry, rather than in purely mechanical interactions between alumina and SiC [79]. However, there exists no general agreement on which mechanism is responsible for the observed changes in mechanical and wear properties of “nanocomposites”, and it remains unclear whether the SiC particles inside the alumina grains or those at the grain boundaries are primarily responsible for these changes.

**Figure 2:**
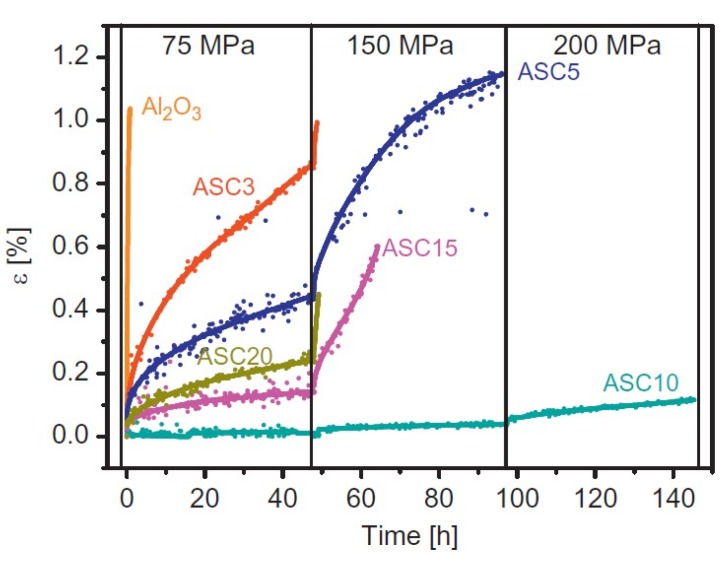
Creep deformation of Al_2_O_3_-SiC (AS) nanocomposites measured at 1350 °C and mechanical load of 75, 150, and 200 MPa. The increase of load is reflected as a break at the stress-strain curve. The number in the sample denomination represents the volume fraction of SiC in the material, *i.e.*, AS5c represents the Al_2_O_3_-SiC nanocomposite with 5 vol% of SiC [88].

As to the high temperature mechanical properties, several observations suggest that the addition of SiC into the Al_2_O_3_ matrix generally increases the creep resistance of Al_2_O_3_-SiC nanocomposites by one to two orders of magnitude in comparison to the monolithic Al_2_O_3_ [80,81]. The mechanisms responsible for the improvement of creep resistance of the Al_2_O_3_-SiC nanocomposites are still under investigation [14,82]. The improvement of creep resistance is generally attributed to the presence of residual stresses, which are created around SiC inclusions in the course of cooling from the temperature of sintering, due to different thermal expansion coefficients of alumina and SiC. The inherent stresses at the alumina–SiC interfaces are compressive, resulting in stronger particle/matrix bonding and inhibition of grain boundary diffusion by intergranular SiC particles and, hence, improved creep resistance [83,84]. In other words, the Al_2_O_3_-SiC interface is much stronger than the alumina–alumina interface, the interfacial fracture energy of an Al_2_O_3_-SiC interface being two times higher than the interfacial fracture energy of an alumina–alumina boundary. Another mechanism contributing to creep resistance is the grain boundary pinning by intergranular SiC particles. As they are engaged with the Al_2_O_3_ grains, the SiC particles rotate, inhibiting the grain boundary sliding and reducing the strain rate in the composite [85,86]. The Al_2_O_3_-SiC nanocomposites with 17 vol% of SiC nanoparticles tested at temperatures up to 1300 °C and at mechanical loads ranging from 50 to 150 MPa, exhibit a creep rate about three orders of magnitude lower and a creep life 10 times longer, than that of the monolithic Al_2_O_3_ under the same conditions. The addition of 5 vol% of SiC to the Al_2_O_3_-based nanocomposites leads to results similar to those mentioned above [87]. However, some authors suggest that higher SiC contents actually decrease the creep resistance due to surface oxidation of the SiC particles. Silica forms an amorphous silicate grain boundary film, which reduces the strength of the Al_2_O_3_-SiC interface bonding. In our previous work we studied the influence of the volume fraction of SiC particles with a mean size of 200 nm on the microstructure and creep behavior of the composites at temperatures up to 1450 °C and mechanical load up to 200 MPa (Figure 2) [88]. The composite with 10 vol% of SiC can withstand stress of 200 MPa at 1350 °C and 1400 °C for 150 h, while the monolithic Al_2_O_3_ reference fails already after 0.8 h at 1350 °C and a load of 75 MPa. The creep resistance of the composites increases with increasing volume fraction of SiC in the concentration range between 3 and 10 vol%. The improvement is attributed to the pinning effect of intergranular SiC particles. At higher SiC contents (15 and 20 vol%), the creep resistance is impaired significantly as the result of microstructure refinement.

### 3.2. Al_2_O_3_-CNT, CNF

Tremendous effort in the last couple of years focused on the research into CNT- or CNF-containing alumina-based nanocomposites was motivated by the extraordinary mechanical properties of both the single- and multi-walled carbon nanotubes, initiating large expectations concerning the improvement of the mechanical properties of polycrystalline alumina. As yet, the results remain controversial. Due to the high aspect ratio of CNT, some improvement of mechanical properties of alumina-based ceramics can be expected at a CNT content as low as 0.01 wt%. However, in many cases much higher CNT contents do not result in any observable improvement. On the contrary, many authors report deterioration of mechanical properties through addition of the CNTs. In order to understand such discrepancies the nature of CNTs, as well as the processing conditions, must be considered. The main problem is a great variety of choice of carbon nanotubes, which differ significantly due to the conditions of their preparation, and subsequent treatment. Different types of CNTs exhibit various levels of mechanical strength, density, and affinity to the ceramic matrix due to differences in their tubular structure, numbers and crystallinity of rolled graphene sheets, number and nature of surface defects, and surface chemistry, just to name only the most important ones [89]. Inappropriate choice of carbon nanotubes or treatment then impairs the mechanical properties of the nanocomposite [36]. This is further complicated by the fact that the understanding of nanostructured composites, and especially the nature of interfacial phenomena between CNTs and the Al_2_O_3_ matrix, which is of crucial importance for mechanical behavior, is far from satisfactory. Recent reports indicate that the grain boundary structure at the CNT–alumina interfaces has a strong influence on mechanical properties [53]. In order to exploit the exceptional elastic properties of CNTs, a strong interfacial bonding at the Al_2_O_3_-CNTs interface is considered vital. An Al_2_OC phase possibly formed by carbothermal reduction of Al_2_O_3_, with good chemical compatibility with both the CNTs and the alumina matrix, has been found to increase the pullout resistance of the CNTs from alumina matrix. This way the high elastic modulus of the CNTs is exploited, bridging the cracks, hindering the crack propagation, and leading to improved fracture toughness [52].

There are also other factors affecting the mechanical properties of CNT-reinforced composites. The first and most crucial one is uneven distribution of the CNTs when an unsuitable dispersion technique is used. Suitability of the dispersion method is defined by the nature of the matrix phase and its surface charges and particle size distribution in the composite suspension, as well as the diameter and length of the used CNTs. The second reason is related to problems with densification, sometimes also due to uneven dispersion of the CNTs. In this case, ceramics with low relative density and poor mechanical properties are obtained despite a high CNT dispersion, irrespective of whether MWCNT or SWCNT is used [90]. The influence of CNT addition on various mechanical properties will be discussed in more detail in the following sections.

#### 3.2.1. Elastic Modulus

The effective elastic modulus of the alumina composites containing up to 1 vol% of MWCNT is usually comparable or slightly lower than that of the monolithic alumina. With increasing volume fractions of the MWCNT, the modulus further decreases [38,55]. The main reason for the observed decrease is the presence of a significant amount of residual porosity: the pores are known to act as a second phase with zero modulus [91]. Another reason cited is the low elastic modulus of the MWCNT. Yu *et al.* [92], report the elastic modulus of the MWCNT at the level of 270 GPa due to imperfections in its structure, and structural defects originating from its treatment. Such damaged nanotubes can then be expected to reduce the elastic modulus of the composites.

#### 3.2.2. Hardness

**Figure 3:**
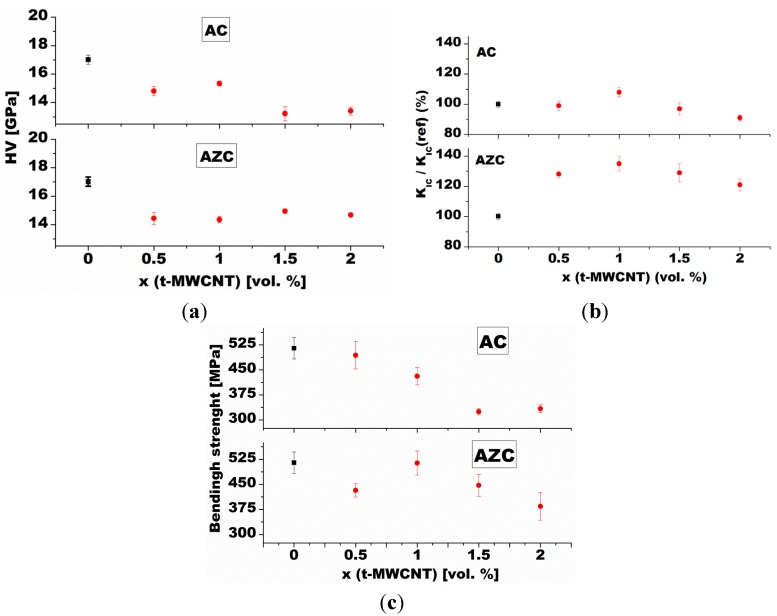
Composition dependence of (**a**) hardness; (**b**) fracture toughness; and (**c**) fracture strength of Al_2_O_3_-MWCNT (denoted AC) and Al_2_O_3_-ZrO_2_-MWCNT (denoted AZC) nanocomposites. Comparison to monolithic alumina reference [95]. Red circles represent the respective properties of nanocomposite with various volume fractions of MWCNT. Black squares represent the same property of the monolithic alumina reference.

Several counteracting influences must be taken into account when evaluating the hardness of CNT-reinforced alumina composites. However, in most reported cases the addition of CNTs results in decrease of microhardness. These results are most often related to the presence of residual porosity, which increases with the CNT content. The trend is further aggravated by uneven dispersion of the CNTs in the matrix, the presence of porous bundles of nanotubes, which act as defects with no load-bearing capacity, and poor cohesion between CNTs and the matrix [93,94]. As a result, a decrease of hardness from 17 GPa in monolithic alumina to 12.5 GPa was observed in the nanocomposite with the addition of a mixture of MWCNT and SWCNT [36]. Other works report monotonous decrease of Vickers hardness with increasing volume fraction of carbon nanotubes, sometimes with small positive deviation from the trend at 1 vol% of MWCNTs (Figure 3a) [95]. The result is attributed to weak interfacial bonding between the MWCNT and the alumina matrix grains. The positive deviation observed at 1 vol% of the MWCNT is attributed to the slightly higher relative density of the composite. The hardness decrease at higher volume fractions of MWCNT is due to the presence of poorly dispersed bundles of MWCNTs, acting as residual porosity in the matrix. These effects can be to a certain extent counteracted by refinement of alumina matrix grains in the final stage of sintering due to pinning action of CNTs at grain boundaries. In fine (and especially submicron) grains, the slip of dislocations is blocked by grain boundaries, resulting in increased hardness of the material. In general, decrease of hardness in the nanocomposites with increasing CNT content is usually attributed to several factors, including the presence of soft phases at the alumina grain boundaries (hardness of MWNTs in the radial direction is 6–10 GPa [89]), poor adherence between the CNTs and the ceramic matrix grains, the lubricating nature of the CNTs, and poor dispersion of CNTs in the alumina matrix, which counteract the influence of microstructure refinement due to the pinning effect of the CNTs at grain boundaries.

#### 3.2.3. Fracture Toughness and Strength

Large interest in the use of carbon nanotubes as a strengthening and toughening agent has been spurred by the work of Zhan *et al.* [96], who reported a fracture toughness of 9.7 MPa·m^1/2^ in a composite with alumina matrix containing 10 vol% of SWCNTs manufactured by spark-plasma sintering. This represents a nearly 300% increase in comparison to monolithic alumina. Since then many attempts have been made to use carbon nanotubes as a toughening agent (Figure 3b) [95,97,98,99]. Most investigations focused on alumina-based composites, using either SWCNTs or MWCNTs, but conclusive demonstration of toughening has not been achieved [100]. The results are generally (but not unambiguously) disappointing for the composites containing MWCNTs, where most authors observed only a marginal increase, or even a decrease of fracture toughness in comparison to monolithic alumina [98,101,102,103,104]. However, several authors reported a significant improvement of fracture toughness in MWCNT–alumina composites [105,106], similar to that achieved by Zhan [96]. Concerning SWCNTs, a few conflicting reports exist [97,107]. Contrary to the results of Zhan [96], several authors report no toughening effect in the Al_2_O_3_ nanocomposites with 10 vol% of SWCNTs, which were as brittle as the monolithic alumina [93]. Despite the contradictions, most authors believe SWCNTs to be more efficient toughening agents than MWCNTs. This is sometimes explained by the fact that MWCNTs exhibit easy sliding between individual graphene sheets in a “sword and sheath” manner, with small ability to carry or transfer loads [108]. Moreover, the MWCNTs have much lower bending strength and stiffness than the SWCNTs, which makes them less efficient reinforcement aids [109]. Yamamoto *et al.* [110], observed initiation of cracks at defect sites in the outer wall of the MWCNTs in external tensile stress field. The cracks propagate through the MWCNTs without any interwall sliding, *i.e.*, the nanotubes simply break. The results suggest that defects detrimental to the load bearing capacity of MWCNTs can by created through thermal damage in the course of high temperature treatment of the nanotubes.

As to the mechanisms responsible for anticipated toughening, even more controversy exists. Most authors agree on the ability of CNTs to induce conventional toughening mechanisms, such as crack bridging and CNT pullout [111,112,113], in some cases combined with crack deflection at CNT–alumina interfaces [96,114]. Some authors also report the weakening of grain boundaries with CNTs, most likely due to formation of CNT agglomerates, difference of thermal expansion coefficients of the CNT and the alumina matrix, and resulting tensile thermal residual stresses across the grain interfaces [107,115,116]. Intergranular fractures characteristic of the CNT-reinforced composites are considered as evidence for such grain boundary weakening [96,105]. However, due to the fact that the addition of CNT usually results in marked refinement of alumina matrix grains (often to sub-micron level) the contribution of crack deflection to fracture toughness is negligible. If the load-bearing capacity of CNT is to be fully utilized, a strong interfacial bonding must exist between the CNTs and the matrix. The friction force between CNTs and matrix is thus the most important factor mediating the energy absorbing role of the CNTs during the crack bridging and pull-out process [105,112]. Tailoring the interfacial strength thus seems an efficient way to increase toughness of the composites. Song *et al.* [37], increased the adhesion between MWCNTs and the alumina matrix by coating the nanotubes with a layer of Al_2_O_3_ nanocrystals, significantly increasing the friction force between MWCNTs and the Al_2_O_3_ matrix grains. Strong interfacial bonding then makes full use of the high elastic modulus and tensile strength of the CNTs, leading to increased toughness of the composite. The toughening action of the CNTs can be then in principle described as follows: The alumina matrix breaks intergranularly, the crack preferentially following the grain boundaries, where weak matrix–CNT interfaces are created due to the CNTs accumulation. The cracked surfaces are then bridged by the CNTs, oriented perpendicularly to the direction of propagating crack, their ends firmly attached in the matrix. The energy dissipation is attributed to the work done by elastic extension of CNTs [105], combined with the work of friction related to pulling out either end of the CNTs from the matrix.

Marginal improvement, or even deterioration, of flexural strength of CNT-reinforced nanocomposites is usually associated with the failure to achieve required dispersion of the CNTs and problems with densification, which result in the presence of residual porosity. Both residual pores and residual bundles of agglomerated nanotubes act as strength defining defects and origins of fracture (Figure 3c) [95].

**Figure 4:**
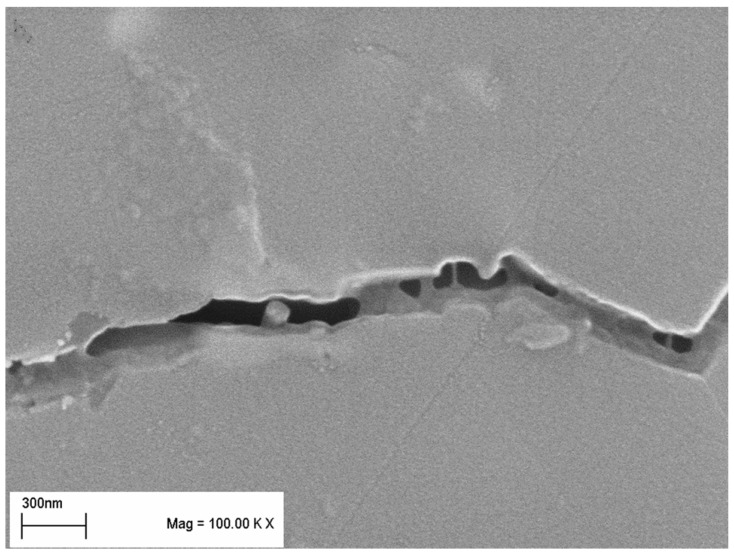
Indication of toughening mechanisms observed in Al_2_O_3_-ZrO_2_-MWCNT nanocomposites [95].

From the point of view of improving the mechanical properties of alumina ceramics, an interesting option is represented by the so-called hybrid microstructure design, *i.e.*, preparation of complex Al_2_O_3_-ZrO_2_-CNT or Al_2_O_3_-SiC-CNT microstructures [60,90]. The addition of as little as 0.01 wt% of MWCNTs to conventionally sintered zirconia toughened alumina (ZTA) ceramics is reported to result in an increase of fracture toughness, with the MWCNT acting as an efficient toughening agent. Similar results observed in ZTA ceramics reinforced by the addition of up to 2 vol% of MWCNT are attributed to synergy effect of transformation toughening with small contribution of crack bridging and CNT pull-out (Figure 4) [95].

In the Al_2_O_3_-SiC-CNT composites, the observed toughening is attributed to the strengthening of grain boundaries and toughening of the alumina matrix by nanosized SiC particles combined with fiber toughening mechanisms from MWCNTs. The incorporation of SiC nanoparticles is also believed to remove residual stresses at the alumina–alumina boundaries, and in matrix grains by generating dislocations around the particles [115,116]. Elimination of tensile stresses strengthens the grain boundaries and impedes the intergranular fracture observed in alumina with added CNTs.

In order to employ excellent elastic properties of carbon nanotubes, and to achieve toughening in alumina matrix composites, the following points are thus crucial: (1) homogeneous dispersion and de-agglomeration of carbon nanotubes in the matrix must be achieved; (2) the composite must be sintered to a high density and the residual porosity eliminated; (3) strong interfacial bonding between CNT and the matrix must be achieved, and (4) high temperature or oxidation damage to the CNTs must be avoided [117].

#### 3.2.4. Tribological Properties

The trends observed in tribological characteristics of CNT-containing alumina-based composites are ambiguous. Some improvement of sliding wear resistance is observed at lower CNT contents, attributed to the grain size effect (refinement of alumina matrix grains with the addition of CNTs) combined with lubrication action of the CNTs (carbon) resulting in marked decrease of friction coefficient. However, despite a sharp decrease of friction coefficient at high CNT volume fractions, the wear losses increase significantly at a CNT content above 10 vol%. This trend is usually explained by deterioration of mechanical properties and increased level of residual porosity due to already discussed difficulties with even dispersion of CNTs and poor cohesion of CNTs with the alumina matrix [58].

#### 3.2.5. Carbon Nanofibers

Carbon nanofibers are often considered as a more readily available and cheaper substitute for carbon nanotubes, with similar capacity for improving both the mechanical and the functional (electric conductivity) properties of alumina-based composites. However, similarly to CNTs, the results are often confusing and contradicting. Generally speaking, the addition of carbon nanofibers leads either to marginal improvement [118] or significant deterioration—by about 40% in comparison to monolithic alumina [119]—of fracture strength. Hardness and fracture toughness are degraded [118]. The disappointing results are usually attributed to the high affinity of the CNFs to form aggregates due to strong van der Waals interactions among them, and, as a consequence, uneven dispersion of CNFs in the composites. The addition of CNFs results in an increase of wear resistance (lower wear rates, decrease of friction coefficient) measured under the conditions of sliding wear using the ball-on-disk technique [120]. The improvement is attributed to the lubricating effect of the CNFs, making the Al_2_O_3_-CNF composites promising candidates for unlubricated tribological applications.

## 4. Functional Properties

### 4.1. Al_2_O_3_-SiC

**Figure 5:**
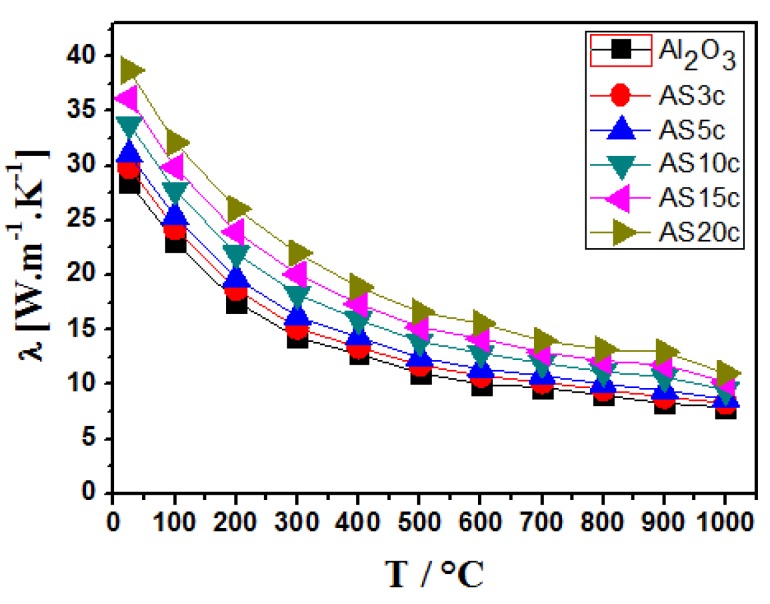
Temperature dependence of thermal conductivity of Al_2_O_3_-SiC (AS) nanocomposites with various volume fractions of SiC. Comparison to monolithic alumina reference [126]. The number in the sample denomination represents the volume fraction of SiC in the material (*i.e.*, AS3c represents the Al_2_O_3_-SiC nanocomposite containing 3 vol% SiC).

Only a few research papers on the functional properties (thermal and electrical conductivity) of Al_2_O_3_-SiC nanocomposites have been published so far [121,122], despite the fact that thermal conductivity is an important parameter in many applications of alumina-based ceramics, including high temperature structural components, refractories for glass and metal production industries, gas radiant burners, wear parts, and cutting tools. In all these applications, the thermal conductivity has to be as high as possible, in order to reduce thermal shock-related failure of the components. Addition of SiC particles can be expected to improve thermal conductivity of the Al_2_O_3_-based nanocomposites due to intrinsically high thermal conductivity of SiC. However, only moderate increase of thermal conductivity has been achieved so far in the alumina matrix composites with SiC inclusions (Figure 5) [122,123]. The relations between the SiC addition and the thermal conductivity of alumina-based composites are complicated, and the influence of interfacial barriers, impurities, and various defects must be considered. The existence of interfacial barriers impairs the conduction of heat by scattering phonons with related increase of the interfacial thermal resistance [124]. It is therefore assumed that the presence of a thermal barrier at the matrix/dispersion boundaries is responsible for the relatively low values of thermal conductivity of this type of composite [125]. In our previous work we investigated the thermal conductivity of Al_2_O_3_-SiC nanocomposites containing 3–20 vol% of SiC particles of two different sizes, 40 and 200 nm. The maximum room temperature thermal conductivity is achieved in the samples containing 20 vol% of SiC particles (38 W·m^−1^·K^−1^), irrespective of the size of the SiC particles, which represents a 35% increase in comparison to the monolithic Al_2_O_3_ reference (28 W·m^−1^·K^−1^). The thermal conductivity decreased with increasing temperature, falling down to 10–C15 W·m^−1^·K^−1^ at 1000 °C (Figure 5) [126].

**Figure 6:**
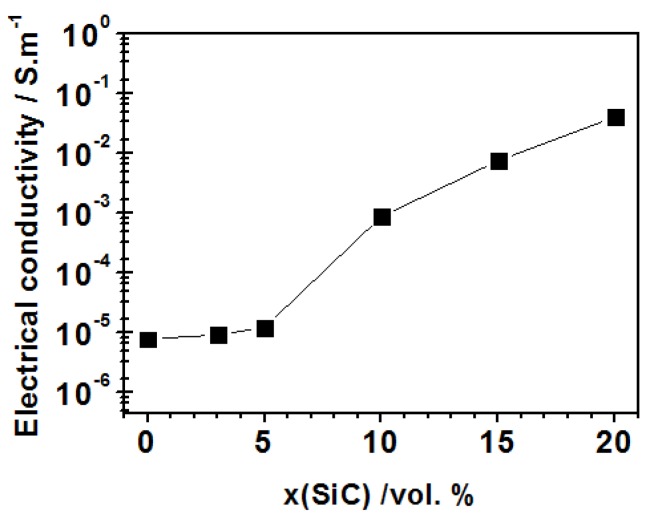
Composition dependence of DC electric conductivity of Al_2_O_3_-SiC nanocomposites with various volume fractions of SiC. Comparison to monolithic alumina reference [126].

Unlike the moderate improvement of thermal conductivity, the electrical conductivity of alumina-based composites can be tailored in a much wider range [121]. The composites reinforced with conductive or semiconductive phases (such as silicon carbide), added in the amount at which they percolate the insulating alumina matrix, have received particular attention. Such electro-conductive or semiconductive ceramics are of special interest in a wide range of industrial applications. In the nanocomposites the electric properties are determined by many critical factors, such as the composition of powder mixtures (the volume fraction of SiC, content of silica as the product of surface oxidation of submicron SiC particles), content of other impurities, and the parameters of the final microstructure of the composite, including the size of alumina matrix grains and the size (micrometer or nanosized) and distribution (intergranular, intragranular or both) of SiC inclusions. The addition of SiC improves DC electrical conductivity, which increases with the volume fraction of SiC [126]. In the composite with 20 vol% of SiC, conductivity of 4.05 × 10^−2^ S·m^−1^ was measured, which represents an increase of four orders of magnitude in comparison to the monolithic alumina reference (7.80 × 10^−6^ S·m^−1^) (Figure 6). The electrical conductivity of the Al_2_O_3_-SiC nanocomposites with the same volume fraction but different size of SiC particles is comparable.

### 4.2. Al_2_O_3_-CNT, CNF

#### 4.2.1. Electric Properties

**Figure 7:**
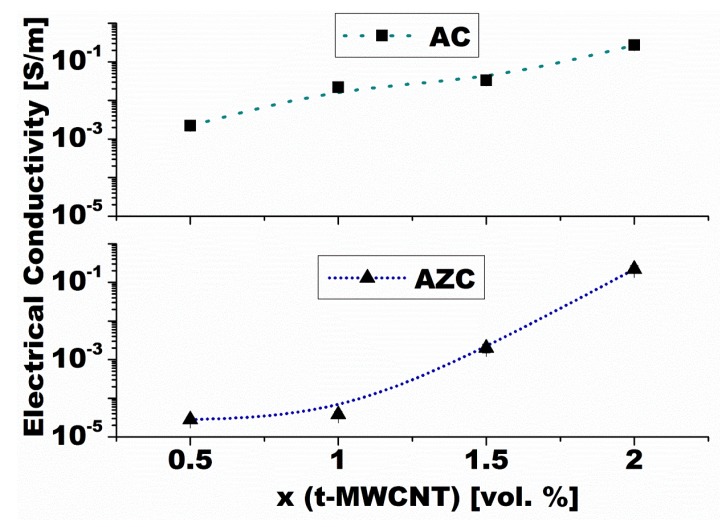
Composition dependence of DC electric conductivity of Al_2_O_3_-MWCNT (material AC) and Al_2_O_3_-ZrO_2_-MWCNT (material AZC) nanocomposites [95].

Due to their intrinsically high electric conductivity, carbon nanotubes (SWCNT or MWCNT) are considered as an ideal candidate for enhancement of the electric conductivity of ceramic materials [127,128] without impairing their mechanical properties. Indeed, the electric conductivity is the only physical property that is, beyond any doubt, markedly improved through the addition of CNTs. The addition of CNFs has am influence on electrical conductivity similar to that of the more expensive MWCNTs [119]. Apart from the high electric conductivity of CNTs or CNFs, an important factor contributing to the electric conductivity of a composite with electrically insulating matrix is the ability of a conductive phase to achieve percolation threshold. The CNTs and CNFs facilitate achievement of the percolation threshold at very low volume fractions, 20 times lower than the percolation threshold achieved in random two-phase composites with micrometer-scale microstructure and isometric morphology of individual components. This low value is attributed to the enormous aspect ratio of MWNTs [128,129]. The DC conductivity near the percolation threshold can be described by power law (Equations (2) and (3)) [130]:

(2) $${\text{σ}}_{\text{m}}={\text{σ}}_{\text{c}}{\left({\text{f}}_{\text{MWNT}}-{\text{f}}_{\text{c}}\right)}^{\text{t}}{\text{ for f}}_{\text{MWNT}}>{\text{f}}_{\text{c}}$$

(3) $${\text{σ}}_{\text{m}}={\text{σ}}_{\text{i}}{\left({\text{f}}_{\text{c}}-{\text{f}}_{\text{MWNTc}}\right)}^{-{\text{s}}^{\prime }}{\text{ for f}}_{\text{MWNT}}<{\text{f}}_{\text{c}}$$

where σ_m_ is the total DC conductivity of the composite; and σ_c_ and σ_i_ are the DC conductivities of the conductive phase and the insulating ceramic matrix, respectively. The symbol f_MWNT_ stands for the volume fraction of the conductive phase, and *f*_c_ is the volume fraction of the conductive phase at which the percolation threshold is achieved. The exponent *t* is the conductivity exponent reflecting the dimensionality of the system, assuming the values 1.33 and 2 for two- and three-dimensional conductivity, respectively, and usually varying between 1.33 and 1.94. However, *t* values lower than 1.94 reflect thermally induced charge hopping transport between loosely connected parts of CNTs rather than the existence of a less than three-dimensional conductive network [131]. Exponent *s*’ is the critical exponent in the insulating region, usually assuming universal values between 0.8 and 1.0. Published experimental data generally indicate an increase of electric conductivity of CNT-containing composites with increasing content of the CNTs. Moreover, the composites exhibit a typical insulator-conductor transition around the percolation threshold. The volume fraction of the CNTs at which the percolation threshold is achieved depends on several factors, including aspect ratio of the used nanotubes, the level of de-agglomeration, and homogenous distribution of the CNTs in insulating matrix. Various authors report the *f*_c_ values ranging from 0.094 to 2.5 wt% of the CNT [61,128,132,133]. The composites typically exhibit a dramatic increase of electric conductivity (at the level of eight orders of magnitude from 10^−12^ S·m^−1^ characteristic for insulating alumina matrix to 10^−4^ S·m^−1^ or more in nanocomposites) when the percolation threshold is achieved. In our previous work we achieved the maximum value of the electrical conductivity at the level of 10^−1^ S·m^−1^ at 2 vol% addition of MWCNTs, which represents an improvement of 11 orders of magnitude with regard to the monolithic alumina reference (Figure 7) [95]. The results exceed by far those reported by Zhou *et al.* [134], who achieved a conductivity of 6.2 × 10^−2^ S·m^−1^ in the composite with the same content of MWCNTs. Such high electrical conductivity is attributed to simultaneous action of two conductive mechanisms: (1) formation of a conductive path through interconnected and percolated network of carbon nanotubes; and (2) evaporation of carbon from MWCNTs, which takes place at the temperature of hot pressing: The carbon deposits at grain boundaries, increasing their electrical conductivity. Further increase in the amount of CNTs beyond the percolation threshold results in only a marginal increase in conductivity, which tends to level off at higher concentrations of carbon nanotubes.

Similarly to DC electric conductivity, the variation of dielectric constant of the composites also follows, in the area close to the percolation threshold, the power law (Equation (4)) [135]:

(4) $${\text{ε}}_{c}={\text{ε}}_{\text{o}}{\left({\text{f}}_{\text{c}}-{\text{f}}_{\text{MWNT}}\right)}^{-\text{s}}$$

where ε_c_ and ε_o_ are the dielectric constants of the composite and the matrix, respectively; *f*_MWNT_ is the volume fraction of carbon nanotubes; *f*_c_ is the percolation threshold, and *s* is the critical exponent.

According to Ahmad *et al.* [128], the dielectric behavior of the CNT-containing nanocomposites can be divided into two categories. In the composites with less than 0.7 vol% of MWNTs, the dielectric constant at room temperature is frequency independent and is defined by dielectric properties of the alumina matrix. As the concentration of MWCNTs approaches the percolation threshold, the dielectric constant increases markedly, assuming a value of about 4600 at a frequency of 1 kHz and MWCNT content of 1.74 vol%. The behavior is attributed to the presence of large number of conducting clusters isolated by thin dielectric layers. Each cluster acts as a minicapacitor: Polarization between the clusters improves electric charge storage. The combination of these factors then contributes to the increase of dielectric constant [136].

#### 4.2.2. Thermal Conductivity

**Figure 8:**
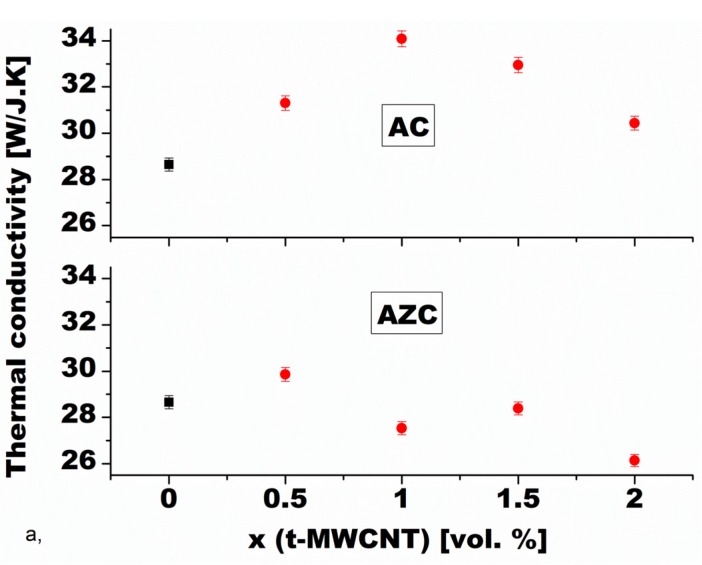
Composition dependence of thermal conductivity of Al_2_O_3_-MWCNT (material AC) and Al_2_O_3_-ZrO_2_-MWCNT (material AZC) nanocomposites [95].

Although there are some works that report increased thermal conductivity in CNT- or CNF-doped alumina-based composites (Figure 8) [95,119,137], the majority of the published data indicates a decrease of thermal conductivity in comparison to monolithic alumina matrix when CNTs are added [129,138]. Such a decrease is understandable if the heat transport mechanism is considered. In ceramic composites with added carbon nanotubes, heat is conducted through propagation of phonon waves [139,140]. Thermal conductivity is then influenced by the sound speed in the composite (related to its elastic modulus), phonon mean free path, and thermal resistance at CNT-alumina interfaces [62]. The thermal conductivity *K*_e_ of CNT-alumina composites can be described using Equation (5) [141]:

(5) $$K_{e}=\;\frac{1}{3}C_{v}ml$$

where *C_v_* is the heat capacity per unit volume; *m* is the speed of sound; and *l* is the phonon mean free path. At temperatures above the Debye temperature, *C_v_* changes very little in comparison to the other two quantities.

The thermal conductivity is therefore controlled by the changes in the phonon mean free path and sound speed. The elastic modulus, and hence the sound speed, is known to decrease with increasing content of CNTs in the composite [62]. The phonon mean free path is influenced by scattering processes related to the arrangement of CNTs and CNT-Al_2_O_3_ interfaces in the composite. The mean free phonon path is reported to decrease with an increasing content of MWCNTs, reaching the minimum values as low as 1 nm, which is much less than the size of matrix grains. Hence, the probability of grain boundary phonon scattering is low [62]. Other authors report much higher values of the phonon mean free path, ranging from 20 to 500 nm [142]. However, even if the grain boundary scattering can be neglected, intertube and intergraphene layer coupling together with scattering at matrix defects markedly contribute to reduction of the phonon mean free path [19,142]. In addition, the MWCNTs dispersed inside the alumina matrix induce interface scattering and further reduce the phonon mean free path.

Apart from reduction of the phonon mean free path, additional mechanisms responsible for decrease of thermal conductivity must also be taken into account. These include high thermal resistance at alumina-CNT interfaces [129] and agglomeration of the CNTs with much lower thermal conductivity than the conductivity of individual CNTs due to intense intertube scattering [143].

## 5. Conclusions

The paper provides a review of alumina-based nanocomposites with added SiC, CNTs, and CNFs, evaluating the efficiency of the additives in terms of their influence on the mechanical and functional properties of the composites. Despite tremendous effort in the last decades focused on improvement of mechanical properties of alumina ceramics, the results remain controversial. Although in some cases significant improvement of mechanical properties is reported (e.g., fracture strength as high as 1000 MPa in Al_2_O_3_-SiC, and fracture toughness of 9.7 MPa·m^1/2^ in Al_2_O_3_-CNT nanocomposites), subsequent work encountered serious problems reproducing the results, achieved more modest improvements, failed to achieve any improvement entirely, or reported deterioration of mechanical properties. The failure to achieve any significant improvement is attributed to a poor understanding of physical and chemical interactions between alumina matrix and the reinforcing phases, problems with de-agglomeration of reinforcing phases and their homogenous distribution in the matrix, and problems related to complete elimination of residual porosity during sintering. Among the mechanical properties, wear resistance is the only one where significant improvement is achieved through the addition of SiC nanoparticles into a polycrystalline alumina matrix. Among the functional properties, electrical conductivity is markedly increased through the addition of highly conductive secondary phases, such as CNTs and CNFs, at concentrations where percolation threshold is achieved, while the thermal conductivity of the nanocomposites is usually impaired by the addition of the second phase.
